# Clesrovimab approval for RSV: a lifesaving opportunity for global infant health equity

**DOI:** 10.1097/MS9.0000000000004792

**Published:** 2026-02-16

**Authors:** Sakshi Kumari, Kamil Ahmad Kamil

**Affiliations:** a Internal Medicine Department, Shimoga Institute of Medical Sciences, Shimoga, India; b Internal Medicine Department, Mirwais Regional Hospital, Kandahar, Afghanistan

**Keywords:** clesrovimab, CLEVER trial, FDA approval, global child health, health equity, infant mortality, LMIC, monoclonal antibody, palivizumab, RSV, SMART trial

## Abstract

The respiratory syncytial virus (RSV) remains the primary cause of acute lower respiratory tract infections and mortality among infants worldwide, with disproportionate deaths in low- and middle-income countries due to limited access to supportive care. The U.S. Food and Drug Administration recently approved clesrovimab, a single-dose monoclonal antibody aimed at protecting infants during their first RSV season. Key phase 2b/3 clinical trials demonstrated clesrovimab’s significant efficacy in reducing RSV-associated hospitalizations by 84.3% and medically attended lower respiratory infections by 60.5%, with a safety profile comparable to existing prophylactic treatments in high-risk pediatric populations. The approval marks a significant advancement for infant RSV prevention in high-income settings, but to effectively reduce the global burden, particularly in resource-limited environments, equitable access and affordability must be prioritized. This intervention offers a vital opportunity to decrease RSV-related morbidity and mortality among vulnerable infants worldwide.

*Dear Editor,*

The U.S. Food and Drug Administration (FDA) recently approved clesrovimab – a single-dose monoclonal antibody designed to protect infants during their first respiratory syncytial virus (RSV) season, the leading cause of acute lower respiratory infections in children globally[1].

Worldwide, RSV causes over 3.6 million hospitalizations and approximately 100 000 deaths in children under the age of five each year. Infants younger than 6 months account for nearly half of these fatalities. Alarmingly, 97% of RSV-related infant deaths occur in low- and middle-income countries (LMICs), where access to supportive medical care such as oxygen or hydration is limited[2].

RSV is a highly contagious respiratory illness that affects individuals of all ages, but it poses the greatest threat to newborns, particularly those born prematurely.

The FDA based its approval of clesrovimab on two pivotal phase 3 trials demonstrating clinically significant reductions in RSV-associated disease severity and hospitalizations. In the Phase 2b/3 CLEVER trial (*N* = 3,614), clesrovimab reduced the incidence of RSV-associated hospitalizations by 84.3% and medically attended lower respiratory infections (MALRI) by 60.5% compared to placebo. Notably, efficacy increased with disease severity[3].

The SMART trial evaluated clesrovimab against palivizumab in high-risk infants, including those born extremely preterm or with congenital heart disease or chronic lung disease[4]. Both groups experienced similar rates of RSV-related illness and hospitalization, and clesrovimab’s safety profile was consistent with findings from the CLEVER trial. Merck reported that efficacy in the SMART trial was supported through pharmacokinetic extrapolation from CLEVER data. While short-term safety outcomes were reassuring, continued post-marketing pharmacovigilance will be essential to monitor rare adverse events and safety in broader, real-world infant populations, particularly in LMIC settings with higher comorbidity burdens.

Clesrovimab may complement existing RSV prevention efforts and reduce the seasonal burden on health systems. However, to truly impact global child health, this intervention must be made available and affordable in LMICs, where the RSV burden is greatest. Equitable access to clesrovimab could significantly reduce hospitalizations and mortality among vulnerable infants especially those under 6 months of age who currently lack access to supportive care. Ensuring timely, broad availability every RSV season can help save countless young lives. However, monoclonal antibody-based interventions are historically associated with high upfront costs, which may pose substantial barriers to widespread uptake in LMICs unless pricing, financing, and procurement challenges are proactively addressed.

In addition, long-term effectiveness data across multiple RSV seasons remain limited, and durability of protection beyond a single season has not yet been fully established. Ongoing follow-up studies and real-world effectiveness evaluations will be critical to inform long-term policy decisions and cost-effectiveness analyses, particularly for national immunization programs in resource-limited settings.

From a global health policy perspective, LMIC adoption of clesrovimab will require advance market commitments, tiered pricing strategies, and early engagement with global procurement mechanisms such as Gavi and UNICEF. Integration into national immunization schedules, alongside maternal RSV vaccination strategies where available, could maximize population-level protection while minimizing delivery costs. Technology transfer and regional manufacturing partnerships may further improve affordability and supply sustainability in resource-constrained settings.

This letter to the editor adheres to the TITAN Guidelines 2025 on transparency, integrity, and the use of AI in medical writing and publishing[5].

## Data Availability

Not applicable.
